# Supplementary material for: The Validity and Reliability of Self-Reported Adherence to Using Offloading Treatment in People with Diabetes-Related Foot Ulcers
Source: Sensors (Basel). 2023 Apr 30;23(9):4423. doi: 10.3390/s23094423 (PMC10181495; doi:10.3390/s23094423)
Supplement: Supplementary file 1 [file sensors-23-04423-s001.zip › sensors-2239868-supplementary.pdf]

Supplementary material file S1

1. Please estimate the percentage of steps you wear the offloading device of all the steps you walk in an average day: Circle **<u>any number on the line</u>** ranging from 0 to 100 to indicate your adherence. 0% means you don’t wear the offloading device in any steps, 50% means you wear the offloading device in half of your daily steps and 100% means you consistently wear the offloading device for every single step”

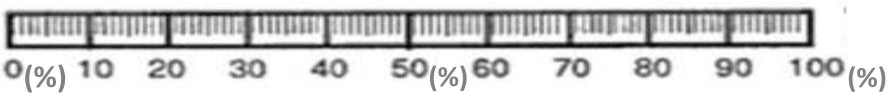

0%=Not wearing the device in any step

50%=Wearing the device in half of daily steps

100%=Wearing the device for every single step
